# Role of Phase-Dependent Dielectric Properties of Alumina Nanoparticles in Electromagnetic-Assisted Enhanced Oil Recovery

**DOI:** 10.3390/nano10101975

**Published:** 2020-10-06

**Authors:** Muhammad Adil, Kean Chuan Lee, Hasnah Mohd Zaid, Takaaki Manaka

**Affiliations:** 1 Department of Fundamental and Applied Sciences, Universiti Teknologi PETRONAS Bandar Seri Iskandar, Tronoh 32610, Perak Darul Ridzuan, Malaysia; hasnamz@utp.edu.my; 2 Department of Electrical and Electronic Engineering, Tokyo Institute of Technology, 2-12-1, O-okayama, Meguro-ku, Tokyo 152-8552, Japan; manaka.t.aa@m.titech.ac.jp

**Keywords:** alumina nanoparticles, dielectric polarization, electrorheological effect, interfacial disturbance, electromagnetic field, enhanced oil recovery

## Abstract

The utilization of metal-oxide nanoparticles in enhanced oil recovery (EOR) has generated considerable research interest to increase the oil recovery. Among these nanoparticles, alumina nanoparticles (Al_2_O_3_-NPs) have proved promising in improving the oil recovery mechanism due to their prominent thermal properties. However, more significantly, these nanoparticles, coupled with electromagnetic (EM) waves, can be polarized to reduce water/oil mobility ratio and create disturbances at the oil/nanofluid interface, so that oil can be released from the reservoir rock surfaces and travelled easily to the production well. Moreover, alumina exists in various transition phases (γ, δ, θ, κ, β, η, χ), providing not only different sizes and morphologies but phase-dependent dielectric behavior at the applied EM frequencies. In this research, the oil recovery mechanism under EM fields of varying frequencies was investigated, which involved parameters such as mobility ratio, interfacial tension (IFT) and wettability. The displacement tests were conducted in water-wet sandpacks at 95 °C, by employing crude oil from Tapis. Alumina nanofluids (Al_2_O_3_-NFs) of four different phases (α, κ, θ and γ) and particle sizes (25–94.3 nm) were prepared by dispersing 0.01 wt. % NPs in brine (3 wt. % NaCl) together with SDBS as a dispersant. Three sequential injection scenarios were performed in each flooding scheme: (i) preflushes brine as a secondary flooding, (ii) conventional nano/EM-assisted nanofluid flooding, and (iii) postflushes brine to flush NPs. Compared to conventional nanofluid flooding (3.03–11.46% original oil in place/OOIP) as incremental oil recovery, EM-assisted nanofluid flooding provided an increase in oil recovery by approximately 4.12–12.90% of OOIP for different phases of alumina. It was established from these results that the recovery from EM-assisted nanofluid flooding is itself dependent on frequency, which is associated with good dielectric behavior of NPs to formulate the oil recovery mechanism including (i) mobility ratio improvement due to an electrorheological (ER) effect, (ii) interfacial disturbances by the oil droplet deformation, and (iii) wettability alteration by increased surface-free energy.

## 1. Introduction

In recent years, nanoparticles (NPs) have been employed as a promising means of improving the characteristics of reservoir and increasing oil recovery, called nano- enhanced oil recovery (EOR) [1,2,3]. Nano-EOR has a few advantages compared to conventional EOR techniques. The small particle sizes of less than 100 nm are thought to move easily through a reservoir rock’s pore throats and be distributed to the pore network locations where they provide an efficient and noticeable change in various ways. The NPs deposition could alter the governing properties of the displacement fluid, including viscosity [4,5], interfacial tension [6,7], dielectric properties [8], alter the rock permeability [9]; or change the wettability of the rock surface [7,10]. Therefore, the size-dependent properties (such as optical, electrical, magnetic, interfacial, and thermo-physical properties) of NPs can be used to aim locations that are challenging for conventional methods to access, such as sensitive downhole sensors [1,11,12].

The rapid progress of nanotechnology has generated different forms of nanoparticles, such as metal-oxides for multi-purpose applications in various fields. At present, nanometals play a key role in multiple areas of physics, chemistry, and materials science [13]. Metal-oxide based nanofluids (NFs) are often employed for heat transfer and thermal conductivity purposes [14,15,16]. The most commonly used metal oxide NPs are Al_2_O_3_, ZnO, TiO_2_, Fe_2_O_3_, MgO, CeO_2_, and ZrO_2_, where each of them possesses distinctive physical and chemical properties [13]. Among these NPs, Al_2_O_3_ is of great interest as an appropriate candidate for innovative applications in EOR such as viscosification, interfacial tension (IFT) reduction, wettability modification, and foam stabilization [3,17,18,19]. Alumina, also known as aluminum oxide, is known to exist in different crystalline transition phases (γ, δ, θ, κ, β, η, χ) which converted to the thermodynamically stable hexagonal α-phase at high temperatures. The transition aluminas have small particle sizes and high surface areas with increased surface activity, making them particularly useful as an EOR agent.

Ogolo et al. observed that alumina NPs of an average size of 40 nm produced a higher tertiary oil recovery (12.5%) compared to other nanometals, when dispersed in brine [20]. Moreover, it was shown that nickel and iron oxide NPs increased the viscosity of brine thereby improving the mobility ratio. In another study, Molnes et al. [21] found that the cellulose nanocrystals also showed an increase in shear viscosity of the aqueous medium, similar to nanoparticles, when dispersed in de-ionized water. It is believed that as shear rates increase, nanocellulose is rearranged to form the organized networks and leads the viscous property to recover [22]. Giraldo et al. reported the change in wettability by the adsorption of Al_2_O_3_-NPs through their coating mechanism [19]. They found that alumina NPs dispersed in the range of 0.01 to 1.00 wt. % along with an anionic surfactant could change the wettability of a strongly oil-wet rock surface into a strongly water-wet surface, as evidenced by a change in residual water saturation S_wr_ from 0.07 to 0.23. Zaid et al. studied the impact of Al_2_O_3_-NPs suspended in an aqueous solution of sodium dodecyl sulfate (SDS) as a stabilizer [23]. Using Al_2_O_3_-NPs of two different types, i.e., sol–gel and commercial Al_2_O_3_, up to 53.5% of the remaining oil is produced. In a two-phase coreflooding process, Hendraningrat et al. reported the potential of alumina on the Berea core plug and demonstrated an increased oil recovery as a result of better nanoparticles adsorption into the pore surface [17,24]. In another study, Mohammadi et al. showed the wettability alteration of Iranian carbonate reservoirs by the application of γ-Al_2_O_3_ NPs [25]. The results depicted that 0.5 wt. % γ-Al_2_O_3_ nanoparticles show a maximum wettability change from oil-wet to water-wet, leading to an increase in oil recovery by 11.25% in a tertiary mode. Wei et al. [22] also believed that nanocrystals, in addition to Al_2_O_3_ NPs, are a promising flooding agent in the near future which showed the sweep volume improvement, emulsification and entrainment as the primary recovery mechanisms for nanocrystal nanofluid flooding. Additionally, Wasan and Nikolov proposed a new view of oil displacement, which investigated the role of disjoining pressure for wetting and dispersing of nanofluids on a solid surface [26]. According to them, a large number of nanoparticles in the wedge film change the flow field structure at a high surface-to-volume ratio, resulting in complex flow structures. Kondiparty et al. [27] also demonstrated that a small contact angle promotes the permeation of the NF film on a solid surface, which restrains the NPs within the film-meniscus area and increases the structural disjoining pressure. Wasan and Nikolov further noted that the dynamics of the contact line are dependent on the combination of the nanoparticle composition, contact angle, and the capillary pressure. A suitable combination of these variables accelerates the spreading of the nanofluid on the solid surface, thereby detaching the oily soil from the substrate [26].

**Figure 1:**
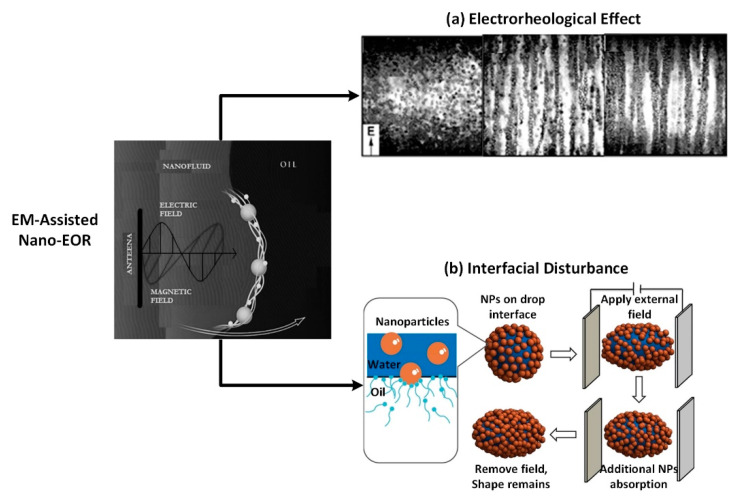
Illustration of oil recovery mechanism of electromagnetic (EM)-assisted nano-enhanced oil recovery (EOR) achieved through (**a**) electrorheological effect as well as (**b**) interfacial disturbance by oil droplet deformation in the presence of dielectric nanoparticles (NPs).

Another way to employ Al_2_O_3_-NPs in EOR processes is by utilizing the electrorheological properties of solid particles suspensions (called electrorheological fluids), such that its rheology will be altered under the presence of electric field [28,29,30,31,32,33,34]. Ahmad Latiff et al. demonstrated an increase of more than 30% in oil recovery with alumina NFs [30]. When subjected to a 60-MHz EM wave, nanofluids showed an increment in their apparent viscosity which in turn enhanced sweep efficiency for greater oil recovery. Haroun et al. also argued that certain metal-oxide nanoparticles could be used to improve the oil production under the applied alternating electrical field, called electrical EOR (EEOR) [31]. The study showed an improvement in the oil production by 9–22% as a tertiary recovery process. Another study demonstrated the capability of EM field to improve the viscosity of the ZnO-NFs injected into the porous medium, thus improving the oil recovery by 23.3% through the electrorheological (ER) effect of the activated ZnO NPs [29]. Our previous work [35] had proposed electromagnetic-assisted nano-EOR that demonstrated an incremental oil recovery of 9–13% original oil in place (OOIP) for 0.1 wt. % ZnO NPs under the application of EM field. Under EM field, the ZnO dispersions not only showed the enhancement of relative viscosity between 5 and 11% at the relatively high shear rate due to an electrorheological (ER) effect, but these NPs were also capable of deforming the oil droplets by showing a noticeable change in IFT and wettability as depicted in Figure 1 [36,37]. The detailed feasibility of EM-assisted EOR in field scale, with an EM source installed inside an injection well can be seen in our previous study [35].

These studies have proven that the oil recovery improved by using ZnO NPs under the application of EM field. However, learning from previous nanofluid experiments, the properties of the displacement fluid are significantly impacted by the choice of the nanoparticles’ material, morphology, concentrations (0.01–1.00 wt. %), and stability in high saline seawater. Therefore, to further understand the effect of EM on oil recovery using metal oxide NPs, this paper has chosen to focus on Al_2_O_3_-NPs because they exist in numerous phases as compared to ZnO NPs, which offers not only different sizes and morphologies but there dielectric behavior changes from one phase to another at a given EM frequency [38]. The dielectric properties are crucial to render NPs as surface-active agents that polarized under an external EM field, resulting in the formulation of possible oil recovery mechanism including (i) the improvement of mobility ratio due to ER effect which increases the viscosity of nanofluids; (ii) the deformation of oil droplets by the polarization of attached NPs, which maximizes the surface area for the additional particle adsorption and therefore resulting in IFT reduction; and (iii) the increase in wettability alteration rate caused by increased surface-free energy. This aim of this investigation is to study the phase-dependent dielectric properties of alumina and their impact on oil recovery mechanism under EM field.

## 2. Materials and Methods

### 2.1. Nanoparticles

The as-prepared Al_2_O_3_-NPs were used for this research. These NPs were prepared earlier by employing the sol–gel auto-combustion approach [39] and then calcinated at different temperatures to achieve a variety of microstructural characteristics and phases. The morphological properties and crystallographic information of these nanoparticles are tabulated in Table 1, corresponding to their calcination temperatures.

### 2.2. Fluids

Sodium chloride (NaCl, Fisher Scientific) was used as a base aqueous phase to prepare brine in deionized water (with σ = 18 MΩ) at 3 wt. % (≈ sea-water concentration). The 0.1 wt. % Al_2_O_3_-NPs were then dispersed in the brine, acting as a base fluid, to prepare the NFs by stirring magnetically for 1 h. Then, sodium dodecylbenzenesulfonate (SDBS, Sigma Aldrich, Kuala Lumpur, Malaysia) was mixed as a stabilizer to the NFs based on the pre-determined optimum concentration of SDBS by using the critical micelle concentration (CMC) method [40]. The solution of HCl/NaOH was used to adjust the pH of NFs to an optimal value (see Table 2) and measured using precise pH meter (FE20-Basic, Mettler Toledo, Switzerland). Afterwards, they were ultrasonicated for an optimum time in an ultrasonic bath (see Table 2) to achieve the required nanofluid.

In order to determine the impact of the different phases of alumina on the oil recovery mechanism, the concentration of Al_2_O_3_-NPs was set at 0.01 wt. % for all the samples. The pH of nanofluids was adjusted to the optimal value (as tabulated in Table 2) in order to enhance the dispersion quality. The high NaCl concentration indicated a change in the isoelectric point (IEP) of the Al_2_O_3_-NPs to higher pH values. This noticeably lowered the NPs stability at higher acidity because of a decrease in the acidic region beyond the IEP, thus, Al_2_O_3_-NFs were prepared in an alkaline environment instead. The density of these samples was also determined using a density meter (DMA 5000, Anton Paar, Graz, Austria). For oil recovery tests, crude oil sourced from Tapis, Malaysia has been used as an oil phase. The 3 wt. % NaCl solution was also employed as a saturation and injection fluid, since brine (≈ sea-water) is produced from oil reservoirs and is readily accessible in offshore fields. These fluid properties are presented in Table 3 under ambient conditions.

### 2.3. Dielectric Measurments

**Figure 2:**
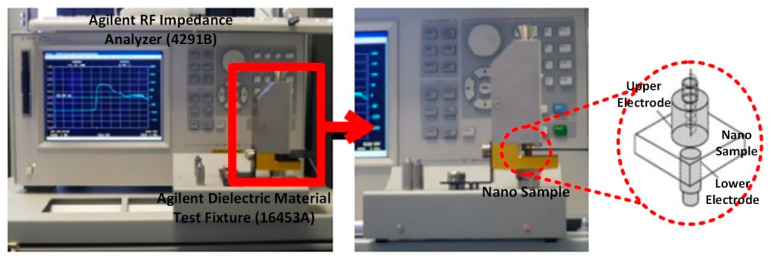
Setup of dielectric measurement of nano-Al_2_O_3_ sample, consisting of radio frequency (RF) impedance analyzer and dielectric fixture with its structure.

The dielectric properties of Al_2_O_3_-NPs, annealed at temperatures between 850 and 1200 °C, were measured using a radio frequency (RF) impedance analyzer (4291B, Agilent, California, USA). In conjunction with Dielectric Material Test Fixture (16453A, Agilent, California, USA), the impedance analyzer was used to determine the dielectric properties at a scale down frequency of 167 and 18.8 MHz (corresponding to a typical Malaysian field-well spacing of 1000 and 3000 m). The nanoparticle pellets, with a diameter of 18mm and a thickness of ~2 mm, were produced by applying an approximately 50 MPa of pressure in a hydraulic press. These pellets are then used to measures dielectric permittivity, including the real (ε′) and imaginary (ε″) part as well as the tangent loss (tan δ). As shown in Figure 2, the configuration of the dielectric fixture comprised of an upper electrode (10 mm in diameter) and a lower electrode (7 mm in diameter), which is identical to the arrangement of parallel plate capacitor. The nanoparticles’ pellets were fastened between the electrodes using an internal spring as a part of the upper electrode, which can also be used to control the applied pressure. The pellets were also covered with silver (paste) to minimize air gap errors.

### 2.4. Viscosity Measurments

**Figure 3:**
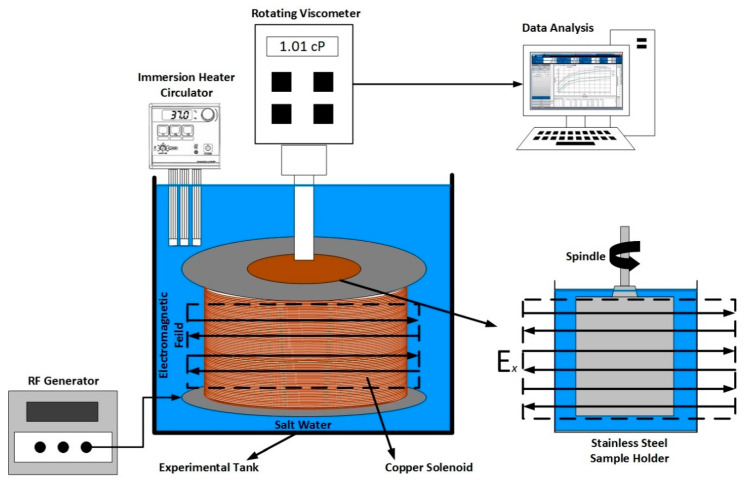
Schematic of viscosity measurement setup of alumina nanofluids under applied EM field and high temperature.

A rotating viscometer (DV−I+, Brookfield, Toronto, Canada) was employed to measure nanofluid viscosity in the share rate between 7.3 and 122.4 s^−1^, considering the instrument limitations. The viscometer was also attached to a custom-made solenoid coil to facilitate the generation of EM field. Two solenoid coils were fabricated with a diameter of 14 and 42 cm, to generate EM at a designed frequency of 167 and 18.8 MHz, respectively. The brief laboratory scale calculation to determine optimum EM frequency can be seen in Appendix A, while the detailed design methodology and experimental validation of EM system can be seen in our previous publication [41]. The schematic illustration of measurement setup is shown in Figure 3; where the viscosities were measured under salt-water as an EM-propagation medium in a laboratory test tank. The temperature of propagation medium was also regulated using an immersion heating circulator (SC-100, Thermo Fisher Scientific, Massachusetts, USA), to investigate the effect of temperature (between 20 and 95 °C) on the viscosity of nanofluids.

The solenoid surrounding the spindle generated the electric field perpendicular to the stainless-steel sample chamber. The UL adapter spindle, which required 16 mL of nanofluid sample, was selected to use with the viscometer. The torque was maintained in the range of 10 to 90% of the maximum torque during the experimental measurement. However, the viscosity of low viscous NFs cannot be determined in this torque range, particularly at a low shear rate. As a result, the low-shear viscosity was measured at a torque value of less than 10% by employing an averaging technique on multiple measurement points to improve accuracy, as suggested by Behi and Mirmohammadi [42].

### 2.5. IFT and Wettability Measurments

**Figure 4:**
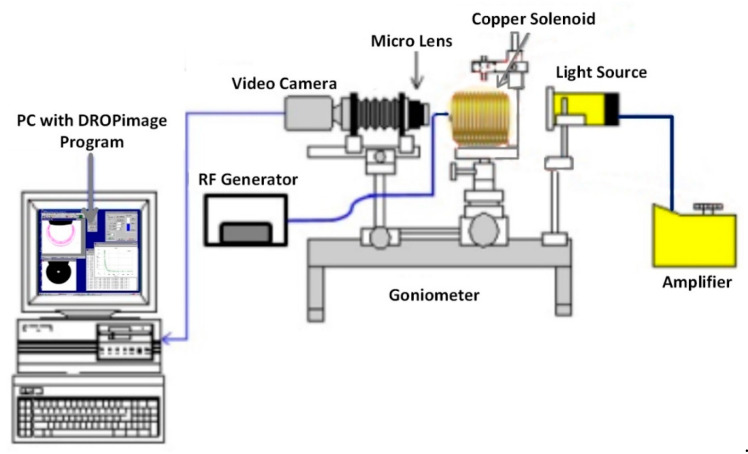
Schematic of goniometer attached with custom-made solenoid coil for the interfacial tension (IFT) and contact angle measurement of alumina nanofluids under electromagnetic field.

The interfacial tension between oil/brine and oil/NF and the three-phase contact angle between solid–NF–oil were measured using the classic approach of sessile drop-shape analysis. These measurements were performed under ambient conditions using a Goniometer (Model 260, Ramé-hart, New Jersey, USA). The measurement setup consists of three major components: (i) a glass plate as a solid surface; (ii) Tapis crude oil as an oil phase; and (iii) Al_2_O_3_-NFs as an aqueous phase. Figure 4 depicted the setup with a glass cell, along with a fabricated solenoid coil, situated between the source of light and the camera. The solenoid with a fixed diameter of 7 cm, because of the limitation of sample size, was attached to the RF generator (33500B, Agilent, California, USA) to generate an EM field at a lower frequency of 18.8 MHz. Air was only used as EM propagation medium, which limits the use of a higher frequency of 167 MHz, leading to a significant loss of EM strength.

**Figure 5:**
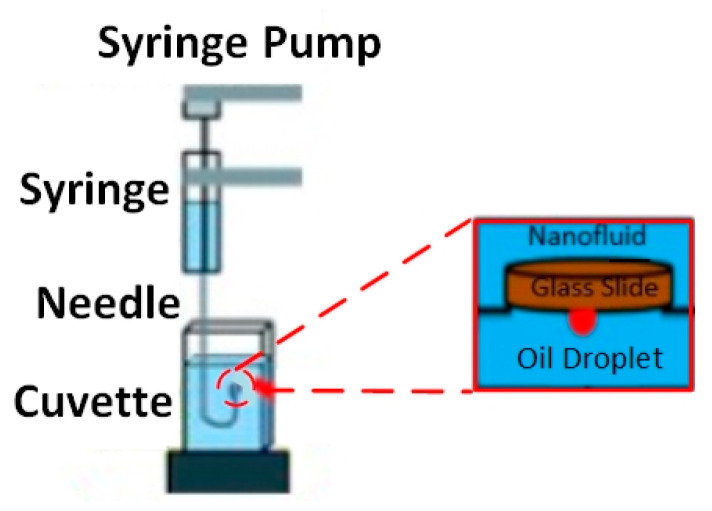
The close-up of measurement setup comprises of a glass slide, crude oil droplet, and Al_2_O_3_-NFs as a solid surface, oil phase and aqueous phase, respectively.

The nano-sample container surrounded by the solenoid coil is separately depicted in Figure 5, where a glass surface representing sandstone is placed on top of the container. Using an inverted syringe, a small drop of crude oil (24 ± 0.2 μL) was positioned under the glass plate. Since oil density is lower than nanofluid density, it was important to place the oil drop under the plate. The camera was manually adjusted to obtain a focused and magnified image of the drop and the surface, which can be seen on the connected computer. The camera was used to capture images once the drop-shape profile reached equilibrium, indicating a steady IFT. DROPimage software then used the side-view profile to measure the IFT and 3-phase contact angle using an image analysis approach.

### 2.6. Sandpack Flooding

**Figure 6:**
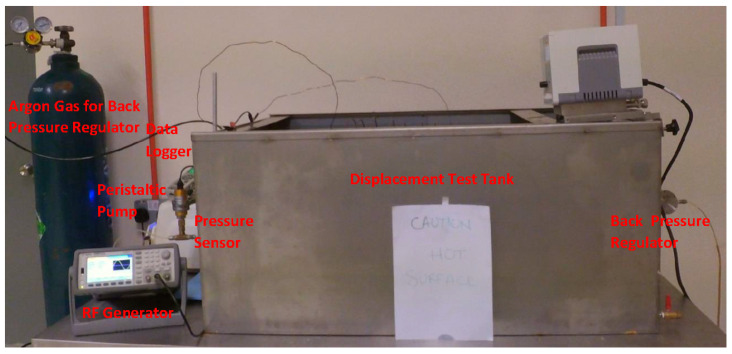
Schematic of experimental setup for nano-Al_2_O_3_ flooding in the presence of EM field, generated by a copper solenoid surrounding the sandpack holder saturated with crude oil.

The acrylic core holder was used as a sandpack, with a diameter and length of 4.6 cm and 30 cm, respectively. For each displacement test, fresh quartz sand (300 to 425 μm mesh size) was packed to achieve the same initial wettability, and then fully saturated with brine at a back-pressure of 50 psi. The porosity and permeability of sandpacks were determined to be in a range of 35–39% and 267–303 mD, respectively. The setup of the two-phase displacement test is shown in Figure 6. A brief process of sandpack flooding is summarized here, while the details can be seen in our previous study [35]. The total of 12 flooding experiments were performed including 4 runs for conventional nanofluid flooding and 4 runs each for EM-assisted flooding at 167 and 18.8 MHz.

**Figure 7:**
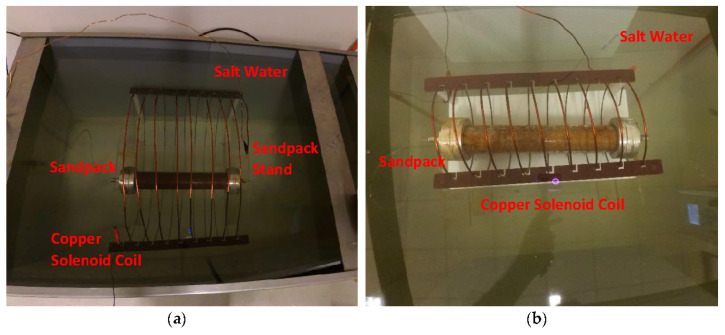
Close-up of test tank comprise of solenoid coil submerged in salt water, while surrounding the sandpack at an experimental frequency of (**a**) 18.8 MHz and (**b**) 167 MHz.

The sandpack was placed inside a tailored solenoid coil in the case of EM-assisted nanofluid flooding. These solenoid coils (as shown in Figure 7) were specifically designed to generate EM field under salt water at a scale down frequency of 167 and 18.8 MHz (corresponding to 1000 and 3000 ft of well spacing). The brine-saturated sandpack was injected with crude oil at 1 cm^3^/min, which was lasted until no further water was recovered to determine the initial water saturation (S_wi_). The injection rate of 1 cm^3^/min is corresponded to 2.83 ft/day and a shear rate of 10 s^−1^, which is consistent with typical shear rates (0.01–10 s^−1^) in most formations [43]. As a secondary recovery process, brine (WF1) was pumped at 1 cm^3^/min to recover the oil, while the system’s temperature and pressure were kept at 95 °C and 50 psi, respectively. The average oil production after WF1 varied from 54.7 to 57% of OOIP, while the residual oil saturation (S_or1_) was recorded as between 42.9 and 45.2% of pore volume (PV). The injection was then proceeded at an identical injection rate (1 cm^3^/min) and pore volume (1 PV) for Al_2_O_3_-NFs as a tertiary recovery process without and with EM field, denoted as NF and EMNF, respectively. In the last step, brine was re-injected (WF2) under the similar conditions as the primary flooding. The oil recovered from flooding was collected by a volumetric graduated cylinder in fractions of 0.1 PV. A differential pressure transmitter within the range of 0 to 10 bar was attached to the sandpack inlet to monitor and record the drop in pressure during the entire flooding process.

## 3. Results and Discussion

### 3.1. Phase-Dependent Dielectric Properties

**Figure 8:**
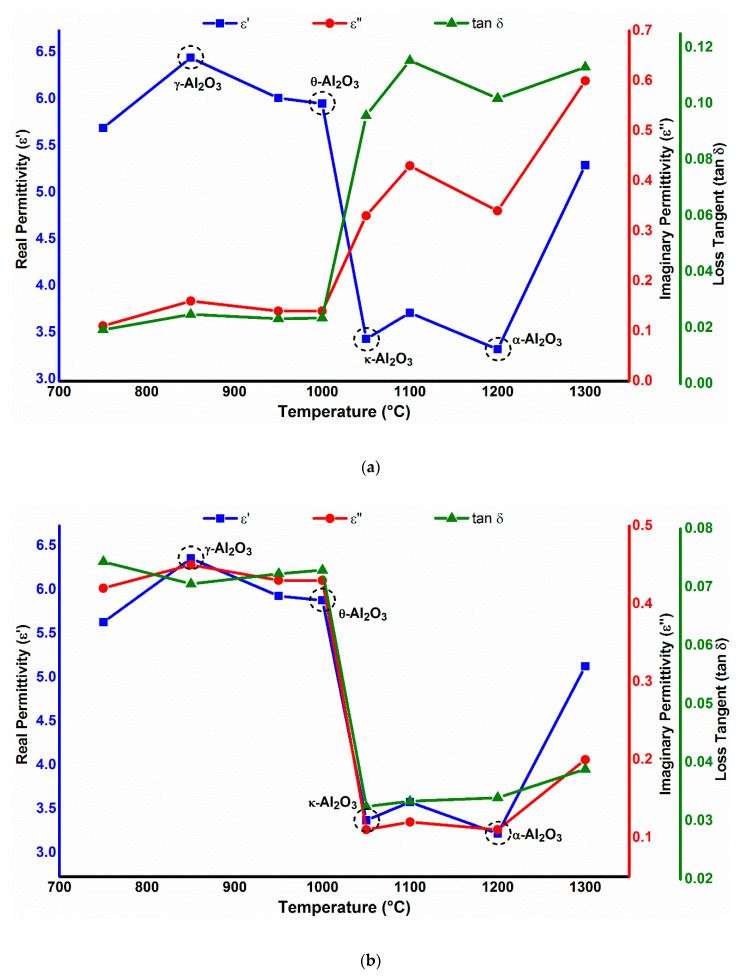
Measured values of ε′, ε″ and tan δ at a frequency of (**a**) 18.8 MHz and (**b**) 167 MHz for different Al_2_O_3_ phases obtained at varying calcination temperature.

Figure 8 represents the dielectric behavior of different phases of Al_2_O_3_-NPs as a function of calcining temperature, at an applied frequency of 18.8 and 167 MHz. A typical trend of increase in dielectric constant can be clearly seen at the applied frequencies with the increase in crystallite size. Between 850 and 1000 °C, higher dielectric values were observed due to the increase in crystallite size. Meanwhile, the dielectric constant experiences a sharp decrease as the particle size decreases from 1050 °C onwards; eventually, due to the particle agglomeration, this increases again at 1300 °C. This can be explained on the basis that polarization is a size-dependent property (more polarization will occur for larger particles).

However, it can be seen clearly from Figure 8 that the dielectric loss of Al_2_O_3_-NPs is not only dependent on the applied frequency, but is also dependent on the phase of Al_2_O_3_ nanoparticles. The dielectric loss value of 0.3 was recorded for stable α-Al_2_O_3_ nanoparticles at a lower frequency of 18.8 MHz, which decreased to lower than 0.1 at a higher frequency of 167 MHz. On the other hand, θ-Al_2_O_3_ NPs showed a smaller dielectric loss of 0.12 at 18.8 MHz, compared to 0.4 at 167 MHz. A large difference in the dielectric loss of alumina samples at very different frequencies was also observed by Vila et al. [44]. The dependence of dielectric loss on phase can be linked to thermal treatment (such as different calcination temperatures and/or time) that alters the intrinsic defect structure by the change in grain orientation. Such intrinsic defects (for example oxygen vacancy or aluminum interstitial) could completely alter the microstructure and possibly destroy or create a dielectrically active dipole, depending on which one is active; therefore, this phenomenon induces an irregular dielectric loss at very different frequencies. With the results obtained, we also did not notice any influence of grain size on dielectric loss. This supports the findings of a previous work in which alumina samples were calcined to increase the grain size under different conditions and no substantial differences were found [44].

### 3.2. Viscosity

**Figure 9:**
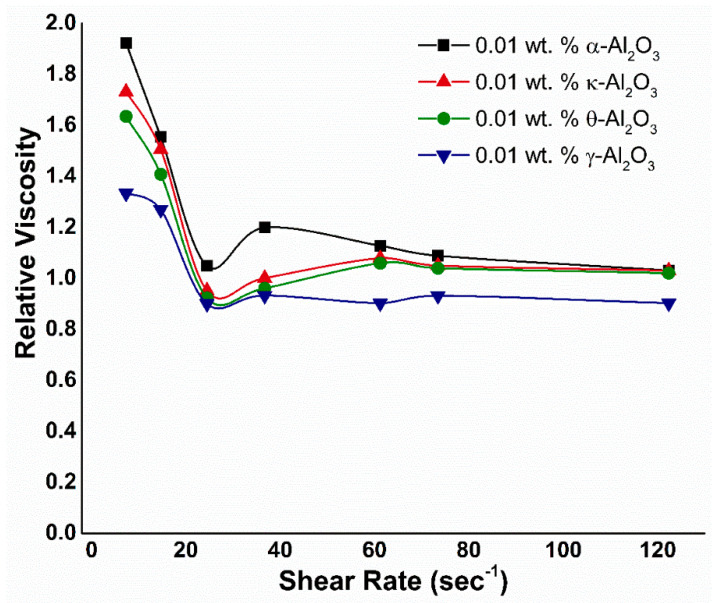
Relative viscosity versus shear rate of alumina nanofluids corresponding to their separate phases.

The relative viscosities for various alumina phases at a constant weight fraction of 0.01 wt. % are plotted against the shear rate in Figure 9. Without an external electric field, it can be observed that all of the nanofluids show a shearing thin behavior. This rheological behavior is often found in suspension systems due to the complex interactions between base fluid and nano-sized materials, such as (a) the disperse and dispersing phase interactions, (b) particle–particle interactions, which determine the degree of particle aggregation, and (c) mechanical interactions of the dispersed particles due to hydrodynamic effects [45]. As the spindle rotates in the nanofluid at low shear rates, the structure of the fluid molecules is aligned steadily in the direction of increasing shear which generates resistance and causes a gradual decrease in viscosity. With the increase in the shear rate, the highest possible shear ordering is obtained and the aggregates are reduced to smaller sizes, resulting in a decrease in friction and therefore viscosity [46]. If the shear rate increases further, there will be no change in the viscosity. Due to the small particle size and large surface area of Al_2_O_3_ (see Table 1), structuring at low shear rates and deformation and restructuring at high shear rates are possible. This is evident from the critical shear value of Al_2_O_3_, suggesting the better resistance at low shear rates. Though the addition of SDBS slightly decreases the interparticle van der Waals interaction in the nanofluid by the shift in IEP (as discussed in Methodology). However, the attraction between the particles still exists due to the identical surface charges. Therefore, this attraction leads to a higher relative viscosity corresponding to the critical shear of Al_2_O_3_-NFs. Hence, all alumina NFs follow the same trend, except γ-Al_2_O_3_ due to poor stability and morphological characteristics (as tabulated in Table 1).

Figure 9 also depicts the dependence of the relative viscosity on hydrodynamic size of water-based nanofluids with Al_2_O_3_ particles. The nanofluids with four particle sizes: 198 nm, 161.5 nm, 109.4 nm, and 342.2 nm were used, corresponding to Al_2_O_3_ phases of α, κ, θ, and γ, respectively. The Al_2_O_3_-NFs represent a similar trend as ZnO NFs: where the viscosity increases with an increase in hydrodynamic particle size. The explanation may be related to the random motion of NPs in the base fluid, which prevents the movement of the base fluid on each other, resulting in an increase in viscosity. Other researchers such as Nguyen et al. [47] and Masoumi et al. [48] established comparable results for Al_2_O_3_-water NFs. Nguyen et al. experimentally studied the impact of the NPs size (36 nm and 47 nm) on the viscosity of Al_2_O_3_-water NFs [47]. According to their observation, for larger particle size of 47 nm, viscosity is noticeably higher than that of 36 nm. On the other hand, Masoumi et al. concluded that the relative viscosity of 28 nm-sized particles is greater than 13 nm-sized Al_2_O_3_ nanoparticles in water at the same concentration [48].

#### 3.2.1. Effect of Temperature

**Figure A1:**
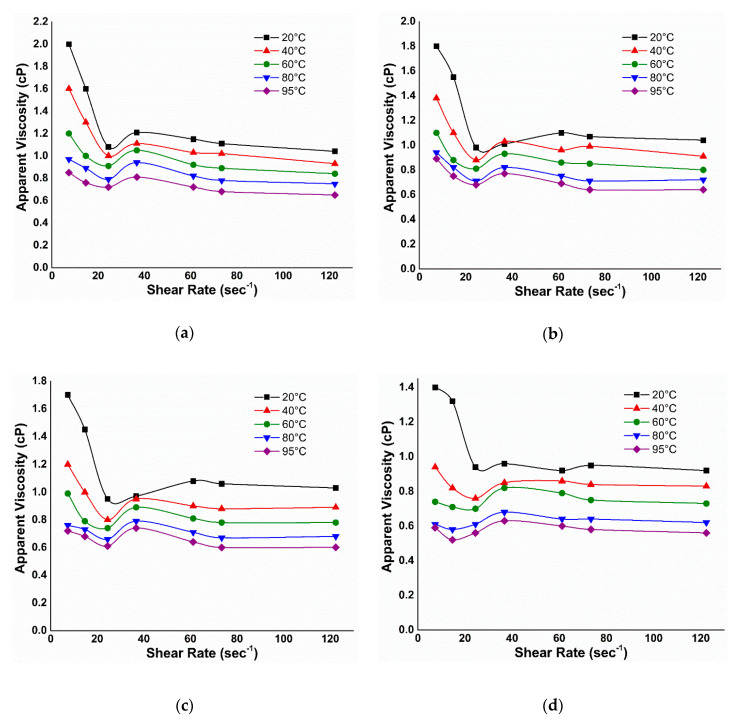
Effect of temperature on apparent viscosity of (**a**) α-Al_2_O_3_, (**b**) κ-Al_2_O_3_, (**c**) θ-Al_2_O_3_ and (**d**) γ-Al_2_O_3_ nanofluids at a fixed concentration of 0.01 wt. %.

The variation in nanofluids’ apparent viscosities with temperature is plotted in Figure A1, ranging from 20 to 95 °C. The results exhibited that the viscosity of nanofluids decreased as the temperature increased, which is due to the fact that with increasing temperature, the adhesion forces of inter-particle and inter-molecular become weak and therefore the apparent viscosity decreases. Jarahnejad et al. studied the impact of temperature, size, and concentration of NPs and the addition of surfactant on the viscosity of water-based alumina NFs [49]. Their results are in accordance with the current study, reporting that the viscosity of NFs decreases due to the temperature increment, similar to the base fluid. Furthermore, the SDBS surfactant used to enhance the nanofluids’ shelf stability is likely to increase their viscosity. Furthermore, these trends indicate that the NFs exhibit shear-thinning behavior between 20 and 60 °C. While the viscosity profile tends to be Newtonian as the temperature further increases, which suggests the complete diminish of adhesion forces. It can also be seen that the viscosity for α-Al_2_O_3_ measures higher than the remaining phases at elevated temperature, which can be related to their corresponding hydrodynamic sizes.

The viscosity of the individual phase of alumina may be the reason for the particle number and surface area per gram for each phase of Al_2_O_3_-NPs, as presented in Table 4. Though the particles are well dispersed in the presence of SDBS, the large particle number of α-Al_2_O_3_ leads to a larger viscosity compare to other phases.

#### 3.2.2. Viscosity under EM Field

**Figure A2:**
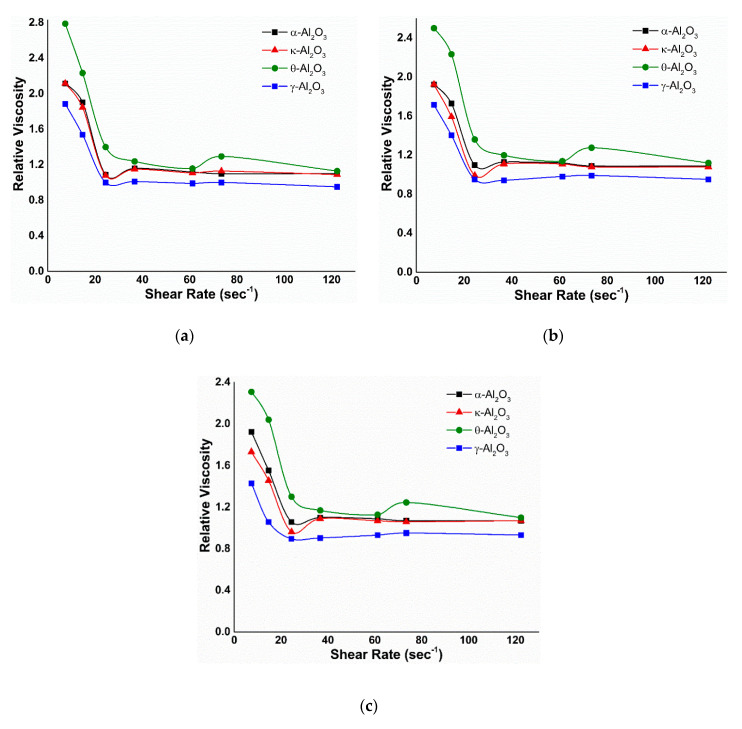
Effect of electromagnetic field at a frequency of 167 MHz and voltages (**a**) 2 V, (**b**) 1.5 V, and (**c**) 1 V, on relative viscosity of alumina nanofluids under salt water as a transmission medium.

Figure A2 shows the electrorheological behavior of alumina NFs under an EM field, generated via a solenoid at varying voltages of 1, 1.5, and 2 V at 167 MHz. The figure portrays the similar shear-thinning behavior as that without the EM field, with an obvious increment in relative viscosity due to ER effect. As the strength of the electric field increases, the increase in relative viscosity was also observed as a result of increased electrostatic interaction between nanoparticles. It is also noticed that the relative viscosity corresponding to the critical shear rate is also increased, compared to no-field relative viscosity. After critical shear, the flow curves of the fluids remain constant, where the shear stress maintains a slight increase as the shear rate increases. Thus, they can sustain a greater ER efficiency in a low shear rate region. The rheological behavior of such fluids is known to be associated with the breaking and reconstruction of chain-like ER structures under the combined application of electric and shear fields [50]. However, the high attenuation as well as the skin depth of salt water, which restricts the propagation of EM waves under high salinity (3 wt. % NaCl), can reduce the relative viscosity [51].

**Figure A3:**
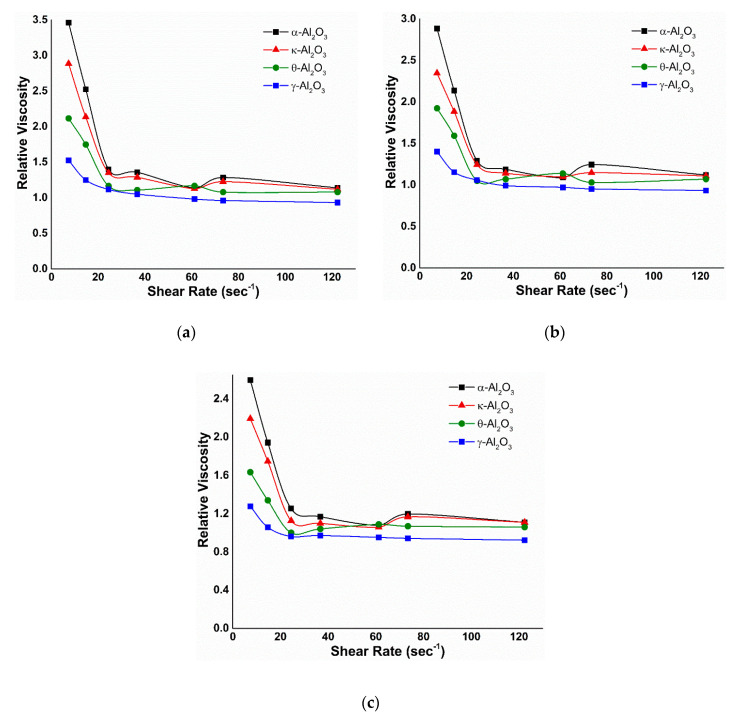
Relative viscosity of different phases of alumina NFs against applied shear rate, in the presence of an EM field under salt water with an applied frequency of 18.8 MHz and applied voltages of (**a**) 3.5 V, (**b**) 3 V, and (**c**) 2.5 V.

For α- and κ-Al_2_O_3_, the electrorheological curve was determined, for which the ER effect was small. Whereas in the electrorheological curve of θ-Al_2_O_3_ NFs, a similar trend to that of α- and κ-Al_2_O_3_ is noticed, but the ER effect becomes prominent at 0.01 wt. % θ-Al_2_O_3_. For γ-Al_2_O_3_ NFs, the relative viscosity does not show a noticeable ER effect, where the ER effects decreased even at lower shear rates. These changes of electrorheological curves in Figure A2 could be explained by the relationship between the shear rate, i.e., the rotational speed of the Al_2_O_3_ nanoparticles, and the polarization rate, i.e., the dipole orientation of the suspended nanoparticles in relation to dielectric loss; a relatively larger dielectric loss ε′′ is crucial to achieve a good ER effect. In the case of α- and κ-Al_2_O_3_, the ε′′ is low consequently the polarization rate is low as well. Chain-like dipoles structures could not build up at higher shear rates due to the very low polarization rate. However, the low polarization rate matches the slow flow condition at a low shear rate to produce a higher ER effect. Meanwhile, at 0.01 wt. % θ-Al_2_O_3_, the high ε′′ value corresponds to the high shear rate flow to create fibril-like structures. Hence, the ER effect is high. In case of applied frequency of 18.8 MHz, the α-Al_2_O_3_ nanoparticles exhibited a high dielectric loss compared to κ- and θ-Al_2_O_3_. Thus, at a larger dielectric loss, a greater relative viscosity is achieved for α-Al_2_O_3_ NFs (Figure A3), which is attributed to a stronger interparticle interaction due to the enhancement of polarization rate and dipolar activity according to the polarization model [52]. Additionally, these figures also showed an enhanced ER effect with the increasing electric current strength, similar to 167 MHz (Figure A2). From the Maxwell–Wagner two-layer condenser [53], it is found that the polarization time constant (τ) of an ER fluid is proportional to the ratio of dielectric constant (ε) and conductivity (σ) of the ER. As the conductivity increases, the electric current moving through the ER fluid increases and the time constant τ decreases, i.e., the polarization rate of the ER fluid increases. This is in accordance with the results showed in Figure A2 and Figure A3.

#### 3.2.3. Effect of Temperature

**Figure A4:**
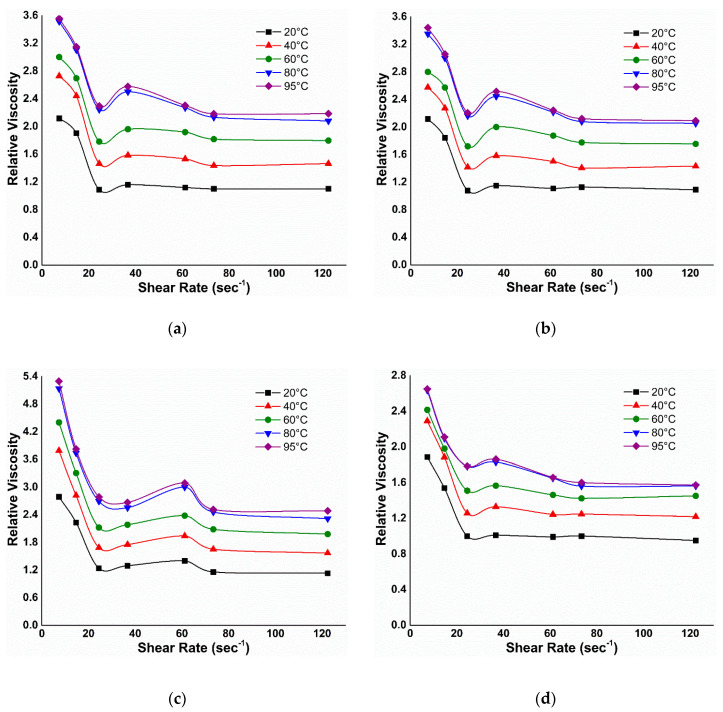
Effect of temperature on relative viscosity of (**a**) α-Al_2_O_3_, (**b**) κ-Al_2_O_3_, (**c**) θ-Al_2_O_3_, and (**d**) γ-Al_2_O_3_ nanofluids under salt water at applied EM frequency of 167 MHz.

In order to observe the electrorheological behaviors of Al_2_O_3_ nanofluids under temperature at 167 MHz, the viscosity of Al_2_O_3_/SDBS NFs versus shear rate was determined at the temperature range between 20 and 95 °C, and the results are plotted in Figure A4. This figure shows that the relative viscosity enhancement at elevated temperature of 95 °C due to the presence of SDBS is clearly evident. Moreover, the ER effect of Al_2_O_3_-NFs particles increases more quickly with the increasing temperature due to the enhanced dipole mobility; consequently, the polarization is promoted more easily. It is also observed that the Al_2_O_3_-NFs shows shear thinning behavior throughout the temperature range, where the shear thinning property is more dominant at low temperature. This shows that at low temperature, the viscosity of base fluid plays a significant role along with interparticle interaction. However, at high temperature, the contribution in viscosity is mainly from the interaction among the nanoparticles. Therefore, the relative viscosity of θ-Al_2_O_3_/SDBS NF under various shear rates of 7.3, 14.6, and 24.4 s^−1^ at 95 °C and 167 MHz is measured to be 5.29, 3.82, and 2.78, respectively, compared to 2.78, 2.23, and 1.23 at 20 °C. The other phases also follow the same trend of relative viscosity with the increasing temperature, except γ-Al_2_O_3_. For γ-Al_2_O_3_, the relative viscosity was decreased for higher temperatures. This is anticipated due to the poor stability of γ-Al_2_O_3_ NFs, which ultimately weakens the interparticle and intermolecular adhesion forces.

**Figure A5:**
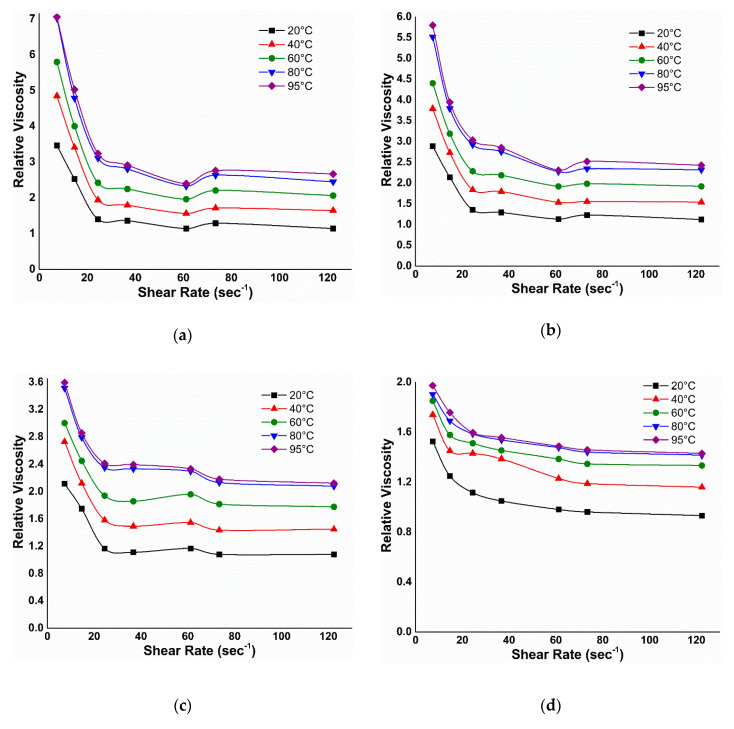
Relative viscosity vs. shear rate of 0.01 wt. % (**a**) α-Al_2_O_3_, (**b**) κ-Al_2_O_3_, (**c**) θ-Al_2_O_3_, and (**d**) γ-Al_2_O_3_ nanofluids under a variety of temperatures, at an applied frequency of 18.8 MHz and 3.5 V.

For 18.8 MHz, the identical electrorheological behavior (as shown in Figure A5) for all the Al_2_O_3_ phases is observed, as of 167 MHz. It can be noticed that the relative viscosity of α-Al_2_O_3_ NF is the highest, which is according to the dielectric properties of different Al_2_O_3_ phases at 18.8 MHz (see Section 3.1). The relative viscosity of α-Al_2_O_3_ NFs at 95 °C; measured to be 7.05 (at 7.3 s^−1^), compared to 5.79, 3.59, and 1.97 for κ-, θ- and γ-Al_2_O_3_, respectively. In particular, the shear rate dependence of relative viscosity still keeps a constant level in the broad shear rate region at high temperatures, further showing the good temperature stability of Al_2_O_3_-NFs.

### 3.3. Interfacial Tension and Contact Angle

**Figure 10:**
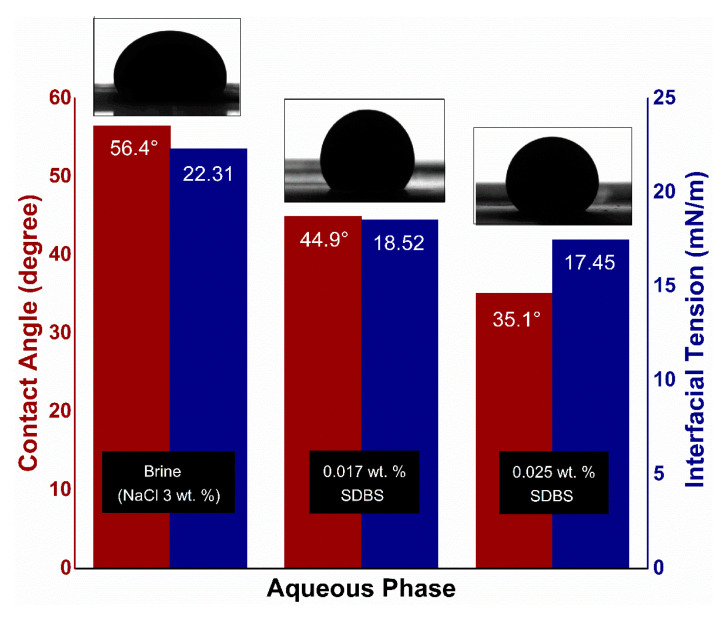
IFT and contact angle measurement of oil droplet against brine and SDBS at room temperature.

Figure 10 depicts the effect of SDBS concentrations, prepared in 3 wt. % NaCl, on IFT and contact angle measurements. As the SDBS concentration increased, IFT declined to 17.45 mN/m, while the contact angle reduced from 56.4° to 35.1° which showed an improvement in the water-wetness of the glass plate.

**Figure 11:**
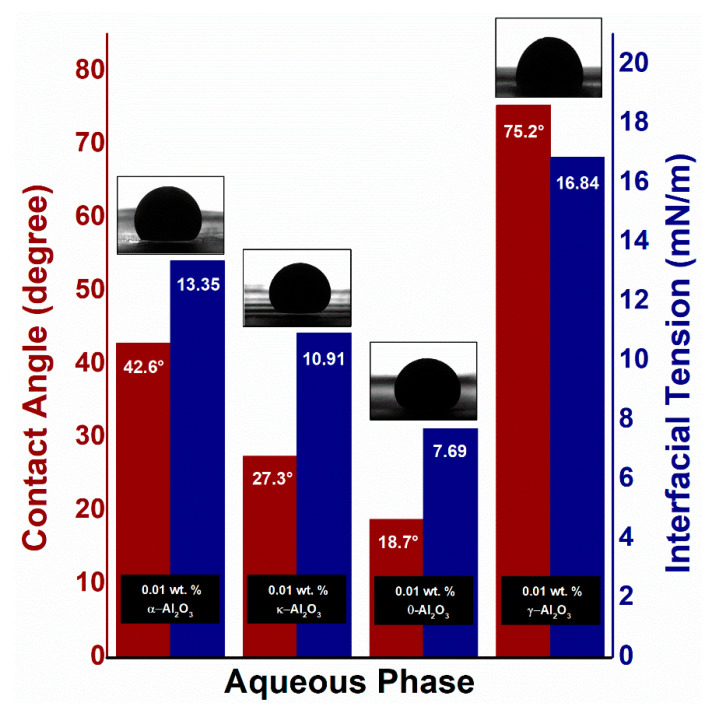
Measured IFT and contact angle of crude oil against the Al_2_O_3_-NFs of different phases together with the captured images.

On the other hand, Figure 11 shows IFT of crude oil alongside SDBS/Al_2_O_3_ NFs at different nano-sizes of alumina phases. Introducing the 0.01 wt. % Al_2_O_3_ NPs into the SDBS/oil system was observed to give lower IFT than SDBS alone. For θ-Al_2_O_3_, the largest decrement of 58.4% was observed in interfacial tension, which is in accordance with its smallest hydrodynamic size, compared to other alumina phases. While the other phases show a decrease of 27.9%, 41.0%, 3.4% for α-, κ-, γ-Al_2_O_3_, respectively.

These findings can be justified on the basis of particle–fluid interactions, which are more effective with a decrease in the distance between nanoparticles and the availability of extra surface area for particle–liquid interactions. Both parameters depend on the concentration of the particles as well as on their size. Smaller particles would give higher surface-to-volume area for the same NPs concentration. It is, therefore, suggested that the reduction in IFT depends on the size of the particle at a given NPs concentration of 0.01 wt. %. Figure 11 also presents the contact angle of crude oil in various aqueous alumina phases. The maximum reduction of 58.2% in the contact angle from 44.9° to 18.74° was measured in the presence of θ-Al_2_O_3_. Whereas the contact angles of 42.76°, 27.34°, and 75.23° were determined for α-, κ-, and γ-Al_2_O_3_ nanoparticles, respectively. These values depicted that nanoparticles have altered the wettability of the quartz plate to a strong water-wet phase due to the NPs adsorption on the plate surface as well as a consequent shift in surface-free energy. Since Al_2_O_3_-NPs possesses weak negative surface charges linked to the poor SDBS adsorption in an alkaline environment, the active nanofluid surface tension along the three-phase contact line appears to change because of adhesive interactions between NPs and quartz surface, resulting in changes in the contact angle. Marrow [54] also observed the large wettability variations of a quartz surface by a monolayer adsorption of polar molecules.

Similarly, as for IFT, the contact angle depends on the size of Al_2_O_3_-NPs and increase as the hydrodynamic particle size increases from 109.4 (θ-Al_2_O_3_) to 342.2 nm (γ-Al_2_O_3_). This change in contact angle could be linked to the surface free-energy of the nanoparticles, which is directly associated with the average particle size as demonstrated in Equation (1) [55]:

(1) $$E_{D}=\;\pi r^{2}\gamma_{wo}\left(1\pm \cos\theta \right)$$

where *r* is the particle radius, *γ_wo_* is the interfacial tension between the two fluids involved and *θ* is the contact angle with the solid surface.

For smaller particles, high surface-to-volume ratio provides a rise in surface-free energy, resulting in a decrease in contact angle. The opposite occurs for larger particles. Results from the research of Vafaei et al. also indicate that the smaller NPs have been more effective in reducing the contact angle of sessile droplets [56]. In addition, Sefiane et al. suggest that there may be two potential underlying mechanisms for improving contact line motion impacted by the introduction of NPs [57]. These mechanisms include improvement due to structural disjoining pressure, or enhancement due to surface adsorption of nanoparticles. Wasan et al. studied the role of disjoining pressure on a solid surface for wetting and spreading of nanofluids [58]. According to him, the presence of a large number of NPs in the wedge film affects the structure of the flow field and results in more complex flow structures. Another study by Kondiparty et al. also argued that a small contact angle helps to spread nanofluid film to a solid surface by restricting nanoparticles in the film-meniscus region and improving the disjoining pressure [27]. Therefore, the greater ratio of surface-to-volume of θ-Al_2_O_3_ NPs offers the smallest contact angle, indicating an increase in the nanofluid’s dynamic spreading.

#### Impact of EM Field

**Figure 12:**
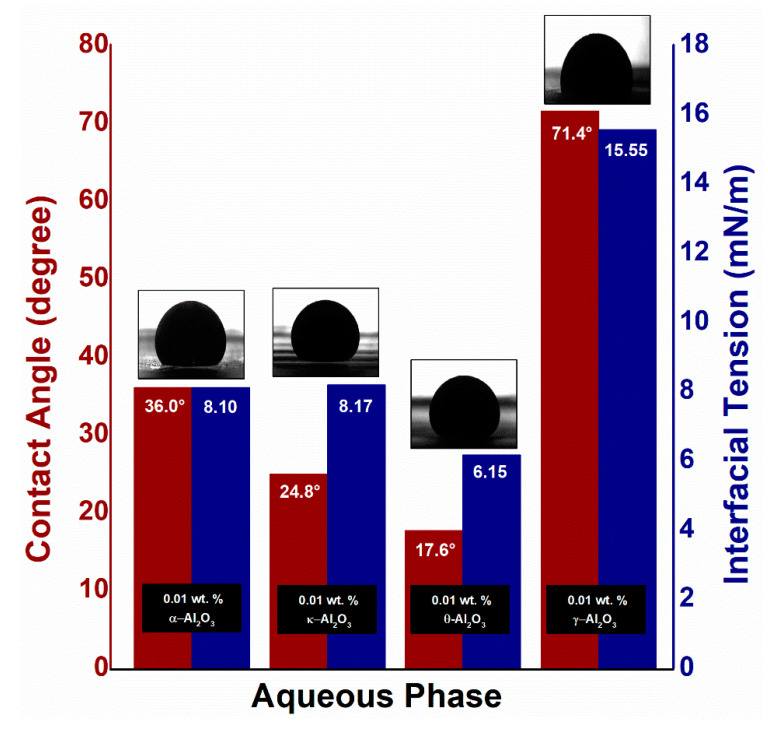
Measurement of oil/Al_2_O_3_-NFs IFT and the glass/Al_2_O_3_-NFs/oil three-phase contact angle under an electromagnetic field.

Figure 12 shows the role of the electromagnetic field on the interfacial tension and contact angle of varying alumina phases at an applied frequency of 18.8 MHz and voltage of 3.5 V. The NPs attached to the oil/NF interface polarized rotationally under an external field, resulting in deformation of the oil droplet shape. This deformation increases the surface area of the oil droplet, permitting more particle adsorption at the interface. As a result, the IFT between the Al_2_O_3_-NF and the oil is reduced. Upon the release of electromagnetic field, the NPs jammed at the oil/NFs interface prevents any surface area alteration by trapping the oil droplet. This reduction in interfacial tension can be linked to the Taylor’s lossy dielectric model [36], which is primarily impacted by the dielectric properties of alumina phases (particularly dielectric loss) at an experimental frequency of 18.8 MHz. As depicted in Section 3.1, the dielectric loss for α- and κ-Al_2_O_3_ is relatively high which provide significant rotational polarization to create a large degree of oil droplet deformation; hence, provide the maximum reduction in interfacial tension from 13.35 to 8.10 mN/m for α-Al_2_O_3_ and 11.89 to 8.17 mN/m for κ-Al_2_O_3_. Meanwhile, the IFT value for both θ-Al_2_O_3_ and γ-Al_2_O_3_ is slightly reduced from 7.69 to 6.15 mN/m and from 16.84 to 15.55 mN/m. This small IFT reduction for θ- and γ-Al_2_O_3_ can be correlated with the weak dielectric properties of NPs at an applied frequency of 18.8 MHz.

The three-phase contact angle also undergoes electrowetting under the EM field [59,60], leading to a contact angle reduction. As presented in Figure 12, the significant decrease is measured in the contact angle for α-Al_2_O_3_ (39.3%) and κ-Al_2_O_3_ (9.1%) related with the motion of polarized dipoles and free charges at three-phase contact line in the presence of electromagnetic field. This causes the Maxwell stress to increase at the oil/NFs interface, which can only be equalized by reducing the curvature of the interface. The contact angle, therefore, decreased from 42.76° to 36.01° for α-Al_2_O_3_, and 27.34° to 24.84° for κ-Al_2_O_3_, in accordance with their dielectric loss.

### 3.4. Nanofluid Flooding

In this section, the effects of different phases of Al_2_O_3_-NPs on the oil recovery were investigated, with and without EM field. The displacement efficiency (*E_D_*, %) at a specific temperature due to nano-EOR was determined and tabulated, using the formula as follows [61]:

(2) $$E_{D}=\;\left[1-\left(\frac{S_{or2}}{S_{or1}}\right)\right]\times 100$$

where *S_or_*_1_ represents residual oil saturation after the injection of brine (waterflooding) and *S_or_*_2_ represents residual oil saturation after nano-EOR. The summaries of these flooding experiments are also tabulated below.

#### 3.4.1. Conventional Nanofluid Flooding

**Figure A6:**
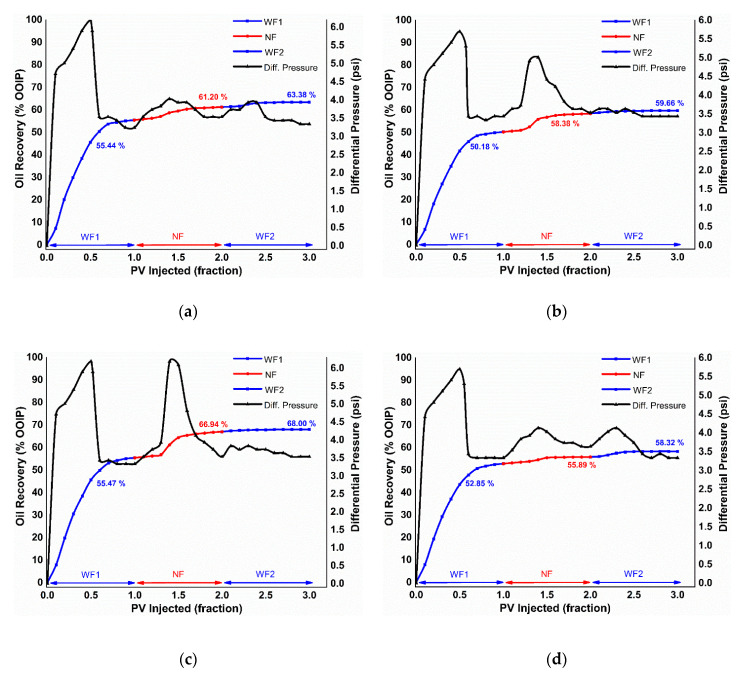
Cumulative oil recovery and differential pressure of (**a**) α-Al_2_O_3_, (**b**) κ-Al_2_O_3_, (**c**) θ-Al_2_O_3_, and (**d**) γ-Al_2_O_3_ nanofluid flooding at a concentration of 0.01 wt. %.

The recovery performance of conventional Al_2_O_3_ nanofluid flooding is presented in Figure A6, and the findings are summarized in Table 5. As depicted in Figure A6, the highest oil recovery of 11.4% of OOIP during the NF sequence was observed for θ-Al_2_O_3_. While 5.7%, 8.1%, and 3.0% OOIP additional oil recovered were observed for the remaining phases of α-, κ-, and γ-Al_2_O_3_, respectively. These results depicted that the additional oil recovery for Al_2_O_3_-NFs flooding is effectively higher than only surfactant flooding (1.6–2.1% OOIP). Based on production profiles, it was also noticed that the incremental oil recovery only started to rise after the injection of 0.4 PV of NFs. This is hypothesized to be due to the NPs adsorption during transport through the porous medium, involving physicochemical interactions between NPs and the sandpack surface [62]. Moreover, the electrostatic interaction between the aqueous solution of NPs and the surface charge of the sand is a critical factor to take into consideration. Al_2_O_3_-based particles (dispersed in SDBS at basic pH) have a weak negative surface charge; thus, when an aqueous solution of Al_2_O_3_-NPs is injected with SDBS, a favorable electrostatic attraction appears to exist between the sand and NPs which causes an increase in oil recovery as compared to the SDBS solution. The use of SDBS surfactant reported a negligible increment in oil recovery (1.6–2.1% of OOIP) after water flooding, in our previous study [35].

The brine was re-injected in the last sequence (WF2) to restore any damage to sandpack properties due to the retention of NPs and to flush out any retaining particles inside the porous matrix likely to reduce the residual oil. As shown in Figure A6, the WF2 scheme led to an increment in oil recovery ranging from 1.06 to 2.4% of OOIP because of the greater ability of Al_2_O_3_-NPs to adsorb into the pore throat, where the particle surface charge plays a significant role in the trapping mechanism [63]. Therefore, the adsorbed alumina particles do not wash out easily during the WF2 sequence. However, the low concentration of 0.01 wt. % NPs used during the flooding tend to have a negligible effect on permeability impairment. Hence, the injected brine continues flowing through the high permeable zone, consequently bypassing the trapped oil.

##### Pressure Drop Profile

The differential pressure during the Al_2_O_3_ nanofluid flooding is presented in Figure A6. During the WF1, the pressure drop increased linearly until water breakthrough at around 0.55 PV, and thereafter generally remained constant at about 3.3–3.2 psi. Once NF injected into sandpack, a sharp increase in the pressure drop was noticed at about 0.4 PV, especially in case of κ- and θ-Al_2_O_3_ (Figure A6b,c). This change in pressure drop may have occurred as a result of NPs adsorption during transport through the porous medium, leading to a change in wettability. This pressure drop association with incremental oil recovery also shows that an increase in oil recovery follows the sudden drop in pressure. Hence, the pressure drop initially increased during the NF sequence, and then decreased once most of the oil was displaced. While in the last sequence of post flushing, the pressure drop decreased slightly for κ- and θ-Al_2_O_3_ (compared to α- and γ-Al_2_O_3_), which represents the small additional oil recovery.

The NPs retention in the porous medium is determined by calculating the residual retention factor (RRF), which is the ratio between the differential pressure after and before the injection of NFs into the porous medium and can be calculated using the following formula:

(3) RRF=∆P2∆P1

where ∆*P*_1_ and ∆*P*_2_ are the differential pressures before, and after nanofluid flooding, respectively.

As presented in Table 6, the RRF values were relatively small for κ- and θ-Al_2_O_3_ NFs; while α- and γ-Al_2_O_3_ depicted a high RRF. The higher the RRF, the higher the possibility of particle retention in the porous medium. Despite the high adsorption capacity of alumina NPs, the RRF value suggests that applying a low concentration of 0.01 wt. % during flooding leads to minimal plugging and the impairment of sandpack properties. However, the higher value of RRF for α- and γ-Al_2_O_3_ can be due to particle aggregation at high temperature, along with the higher tendency of particle adsorption. During the WF2 cycle, the RRF value only slightly reduced for alumina NFs; this suggests the presence of retained particles. The particle retention during transport of Al_2_O_3_-NPs in porous media was also observed in the previous study [64].

#### 3.4.2. EM-Assisted Nanofluid Flooding

**Figure A7:**
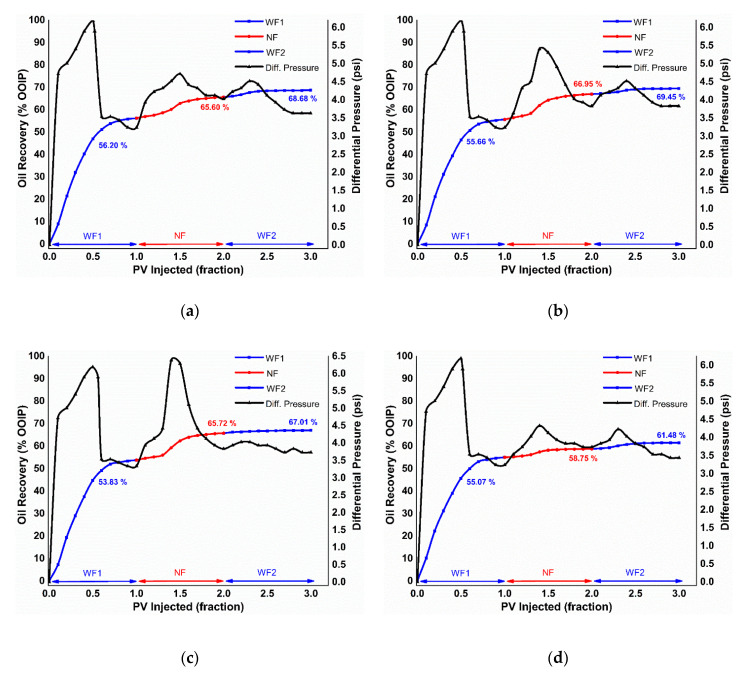
Cumulative oil recovery and differential pressure of EM-assisted nanofluid flooding at an applied frequency and voltage of 18.8 MHz and 3.5 V for (**a**) α-Al_2_O_3_, (**b**) κ-Al_2_O_3_, (**c**) θ-Al_2_O_3_, and (**d**) γ-Al_2_O_3_ nanofluids.

**Figure A8:**
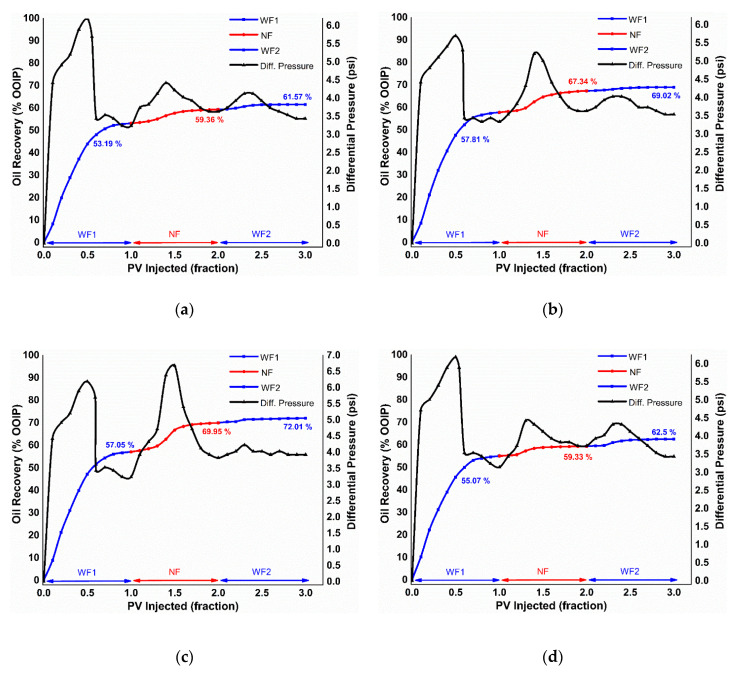
Oil recovery performance vs. differential pressure as a function of fraction of injected PV for EM-assisted nanofluid flooding of (**a**) α-Al_2_O_3_, (**b**) κ-Al_2_O_3_, (**c**) θ-Al_2_O_3_, and (**d**) γ-Al_2_O_3_ nanofluids at an EM-frequency of 167 MHz and applied voltage of 2 V.

The production profiles of EM-assisted Al_2_O_3_ nanofluid flooding are depicted in Figure A7 and Figure A8 at 18.8 and 167 MHz, respectively. The summarized result of displacement tests is also tabulated in Table 7. The results show that the additional oil production for EM-assisted nanofluid flooding is effectively higher than only nanofluid flooding. In comparison to conventional nanofluid flooding, the biggest percentage increase of 38.65% in oil recovery for α-Al_2_O_3_ was observed, under EM field at 18.8 MHz and 3.5 V. While 31.73%, 6.29%, and 29.2% increases in oil recovered were measured for remaining phases of κ-, θ-, and γ-Al_2_O_3_, respectively. On the other hand, the highest increase of 12.9% OOIP (11.1% increase) was recorded for θ-Al_2_O_3_ NFs at the applied frequency of 167 MHz. This increment in recovery from EM-assisted nanofluid flooding is dependent on dielectric behavior (especially dielectric loss) of Al_2_O_3_-NPs. However, the dielectric loss is independent of frequency for Al_2_O_3_ nanoparticles, but depends on its morphology including phase, size (as described in Section 3.1).

The increase in additional oil recovery due to EM field can be justified by three recovery mechanisms including (1) the dielectric polarization of NPs induces the oil droplet deformation which in turn allows the adsorption of more nanoparticles due to an increase in surface area and therefore reduces the interfacial tension; (2) the decrease in wettability by an increase in surface free energy, leading to an increase in structural disjoining pressure; (3) the enhancement in mobility ratio by the increase in the viscosity of Al_2_O_3_-NFs due to ER effect. At 18.8 MHz, the noticeable increase in oil recovery for α- and κ-Al_2_O_3_ (compared to conventional nanofluid flooding) can be associated with the decrease in IFT and wettability as presented in Figure 12. The IFT value reduced from 13.35 to 8.10 mN/m and 10.91 to 8.17 mN/m for α- and κ-Al_2_O_3_ NFs, respectively, while the contact angle was measured to be 36.01° and 24.84° at an experimental frequency of 18.8 MHz. Similarly, the high-temperature viscosity ratio under electromagnetic field shows a good decrement of 0.97 from 2.55 for α-Al_2_O_3_; and 1.2 from 2.49 for κ-Al_2_O_3_ NFs. These visible changes in NFs properties can be related to the high dielectric loss of α- and κ-Al_2_O_3_ at 18.8 MHz. Due to large dielectric loss of α- and κ-Al_2_O_3_ nanoparticles, the achievable polarizability of the nanofluid is enhanced, which increases the additional oil recovery in response. In the case of θ- and γ-Al_2_O_3_, the dielectric loss as well as the polarization rate is relatively low at 18.8 MHz; which consequently provide a slight increase in additional oil recovery. This minimal increase in oil recovery for θ- and γ-Al_2_O_3_ NFs can be related to the small change in IFT/wettability, along with viscosity ratio due to the poor dielectric properties of NPs.

During the post-flush, α- and γ-Al_2_O_3_ NFs shows a slightly more incremental oil recovery of 2.2–3.0% OOIP and 2.7–3.0% OOIP, respectively. Since, α- and γ-Al_2_O_3_ shows particle aggregation at high temperature, along with the higher tendency of particle adsorption. This shows that the injected Al_2_O_3_-NFs have somewhat obstructed the water channels of high permeability, not only because of the aggregated particles, but also because of the ER effect, which ultimately reduces the mobility of the aqueous phase. As a result, the subsequently injected brine is pushed to the bypassed zone and results in a relatively high sweep efficiency compared to conventional nanofluid flooding.

##### Pressure Drop Profile

The differential pressure profile during the EM-assisted Al_2_O_3_-NFs flooding is presented in Figure A7 and Figure A8 at 18.8 and 167 MHz, respectively. These profiles show a clear connection of pressure drop with improved oil recovery, which suggests that the rise in differential pressure is accompanied by a greater mobility of crude oil. In the presence of EM field at 18.8 MHz, the highest-pressure drop was recorded to be 9.6 psi during the 1 PV injection of α-Al_2_O_3_ nanofluid. This increase in pressure drop can be attributed to the (1) particle aggregation at elevated temperature; and (2) formation of a chain network of nanoparticles due to ER effect. The high pressure drop also suggests the increase in apparent viscosity of Al_2_O_3_-NFs, which subsequently mobilize the crude oil by reducing the mobility of the aqueous phase. As a result, the pressure gradually builds up due to the effective blockage of the water channel, and then falls sharply as the differential pressure overpowers the capillary and the viscous forces enforced by the nanofluids [65]. In the case of 167 MHz, the injection of θ-Al_2_O_3_ nanofluid produces a greater pressure drop profile corresponding to the increased oil recovery among the other phases. Due to the smaller hydrodynamic size of θ-Al_2_O_3_, the role of particle aggregation, as well as ER effect, is insignificant in pressure drop, compared to the alteration of IFT/contact angle. This change in IFT/contact angle may have achieved by the NPs adsorption during transport through the porous medium, leading to a high differential pressure. Whereas during the secondary water flooding, the pressure drop decreased gradually for θ-Al_2_O_3_ at 167 MHz (compare to α-Al_2_O_3_ at 18.8 MHz), which represents the small additional oil recovery.

The RRF value, as tabulated in Table 8, also shows an obvious increment with the interaction of particle chains of α-Al_2_O_3_ at 18.8 MHz in the porous medium. The retention of these particles eventually allows the post-flush to enter the unswept zone, which shows a slight increment in differential pressure. While at 167 MHz, the pressure drop initially increased during the NF sequence due to the high adsorption capacity of θ-Al_2_O_3_ NPs, and then decreased once most of the oil displaces. Under an EM field, the low concentration of 0.01 wt. % θ-Al_2_O_3_ NFs during flooding shows a wettability alteration that leads to an increase in pressure drop (caused by structural disjoining pressure). However, at this low particle concentration, only a minimal plugging and sandpack properties impairment is depicted, corresponding to smaller RRF value. Due to the high adsorption capacity of alumina NPs only a slight decrease in pressure drop including the RRF value is observed, during the WF2 sequence (compared to NF). This suggests the presence of retained particles as observed in the conventional nanofluid flooding.

## 4. Conclusions

A comprehensive EOR study was performed to determine the effect of alumina phases on oil recovery mechanism for conventional and EM-assisted nano-EOR by evaluating the relationship between the viscosity ratio (crude oil/NF), IFT (crude oil/NF), and contact angle (crude oil/NF/glass) of Al_2_O_3_-NFs. It was concluded that the oil displacement during the conventional nanofluid flooding depends on the hydrodynamic size of alumina nanofluids, where the interfacial tension and contact angle reduces with the decrement in particle size. These observations are in accordance with and comparable to the improved oil production from the conventional θ-Al_2_O_3_ nanofluid flooding, which exhibits the highest incremental oil recovery (11.46% OOIP) as well as displacement efficiency (25.75%). Meanwhile, the additional oil recovery during EM-assisted nanofluid flooding is mainly dependent on the dielectric loss corresponding to the alumina phase at a given EM frequency. The θ-Al_2_O_3_ NPs shows a greater total oil recovery (72.01% OOIP) at 167 MHz, compared to α-Al_2_O_3_ NPs (61.57% OOIP) and κ-Al_2_O_3_ NPs (69.02% OOIP) due to high dielectric loss of θ-Al_2_O_3_ NPs to achieve greater ER effect as well as interfacial disturbance. On the other hand, the good dielectric properties of κ-Al_2_O_3_ NPs at 18.8 MHz resulted in relatively high total oil recovery of 69.45% OOIP compared to 67.01% OOIP for θ-Al_2_O_3_ NPs. During the post flush, a slight incremental oil recovery of 2.2–3.0% OOIP was observed for α- and γ-Al_2_O_3_ NFs due to their high tendency of adsorption in porous media as well as the formation of large particle aggregates at high temperatures. However, the low particle retention of κ-Al_2_O_3_ and θ-Al_2_O_3_ in the porous medium was determined from the evaluation of differential pressure, suggesting that applying a low concentration of 0.01 wt. % during flooding leads to a minimal plugging despite the high adsorption capacity of alumina NPs. The role of wedge mechanism for oil droplet release under EM field is also crucial, which provides the opportunities for future investigations, both experimentally and theoretically. Overall, this study reveals an innovative way to tailor the different phases of alumina NPs for EOR at a high reservoir temperature.

## Figures and Tables

**Table 1:** Experimental parameters used in the synthesis of silica NPs.

Calcination Temperature (°C)	Crystallite Size (nm) ± 2	Lattice Parameter (nm) ± 0.01	Crystal Structure	Crystallinity (%)	Porosity (%)	Surface Area (m^2^.gm^−1^)	Denotation
850	94.3	a = 3.93 b = 3.93 c = 3.93	Cubic	72.2	32.9	34.5	γ-Al_2_O_3_
1000	64.9	a = 3.93 b = 3.93 c = 3.93	Monoclinic	96.2	41.0	43.3	θ-Al_2_O_3_
1050	55.1	a = 3.93 b = 3.93 c = 3.93	Orthorhombic	96.4	48.3	52.4	κ-Al_2_O_3_
1200	25.0	a = 3.93 b = 3.93 c = 3.93	Hexagonal	98.8	50.3	121	α-Al_2_O_3_

**Table 2:** Optimal stability parameters for the preparation of stable alumina nanofluids, containing the pH, SDBS concentration and ultrasonication time period.

Nanofluid	pH	SDBS Concentration (wt. %)	Ultrasonication Period (min)
γ-Al_2_O_3_	8	0.025	90
θ-Al_2_O_3_	10	0.017	90
κ-Al_2_O_3_	10	0.017	90
α-Al_2_O_3_	8	0.017	90

**Table 3:** Fluid properties under room temperature.

Fluid	Concentration (wt. %)	Properties		
		Density (g.cm^−3^)	Viscosity at 100 rpm (cP)	pH
Crude oil	–	0.8021	7.50	–
Brine (NaCl)	3	1.0197	1.01	6.76
SDBS	0.017	1.0190	1.01	5.25
	0.025	1.0194	1.02	5.16
γ-Al_2_O_3_	0.01	1.0170	0.92	5.03
θ-Al_2_O_3_	0.01	1.0115	1.03	4.62
κ-Al_2_O_3_	0.01	1.0191	1.04	4.62
α-Al_2_O_3_	0.01	1.0196	1.04	4.59

**Table 4:** Specific surface area and particle number of different phases of alumina nanoparticles per 0.01 wt. % nano dispersion.

Nanoparticles	Specific Surface Area (m^2^·g^−1^)	Particle Number per Gram	Particle Number per 0.01 wt. %
α-Al_2_O_3_	121.2	61756296	617562
κ-Al_2_O_3_	52.40	5494017	54940
θ-Al_2_O_3_	43.39	3279527	32795
γ-Al_2_O_3_	34.56	1237326	12373

**Table 5:** Summary of conventional Al_2_O_3_ nanofluid flooding at 95 °C.

Initial Water Saturation (Swi) (% Pore Volume (PV))	Oil Recovery (% OOIP)			Displacement Efficiency (*E_D_*) (%)	Total Oil Recovery (% OOIP)	Flooding Case
	WF1	NF	WF2			
29.75	55.44	5.76	2.17	12.94	63.38	α-Al_2_O_3_
28.62	50.18	8.19	1.27	16.45	59.66	κ-Al_2_O_3_
30.55	55.47	11.46	1.06	25.75	68.00	θ-Al_2_O_3_
26.79	52.85	3.03	2.42	6.43	58.32	γ-Al_2_O_3_

**Table 6:** Residual retention factor for Al_2_O_3_ nanofluid flooding.

Flooding Case	Residual Retention Factor	
	NF	WF2
α-Al_2_O_3_	1.090	0.944
κ-Al_2_O_3_	1.028	0.972
θ-Al_2_O_3_	1.058	1.00
γ-Al_2_O_3_	1.088	0.918

**Table 7:** Summary of EM-assisted nanofluid flooding for different phases of Al_2_O_3_ at the applied frequency of 18.8 and 167 MHz.

Applied EM Frequency (MHz)	Initial Water Saturation (Swi) (% PV)	Oil Recovery (% OOIP)			Displacement Efficiency (*E_D_*) (%)	Total Oil Recovery (% OOIP)	Flooding Case
		WF1	NF	WF2			
18.8	28.62	56.20	9.39	3.08	21.45	68.68	α-Al_2_O_3_
	29.44	55.66	11.28	2.50	25.46	69.45	κ-Al_2_O_3_
	29.75	53.83	11.89	1.29	25.76	67.01	θ-Al_2_O_3_
	29.44	55.07	3.67	2.73	8.17	61.48	γ-Al_2_O_3_
167	28.62	53.19	6.16	2.21	13.17	61.57	α-Al_2_O_3_
	29.44	57.81	9.53	1.67	22.59	69.02	κ-Al_2_O_3_
	29.75	57.05	12.90	2.05	30.04	72.01	θ-Al_2_O_3_
	29.15	57.57	4.12	3.06	9.73	64.77	γ-Al_2_O_3_

**Table 8:** Calculated residual retention factor for EM-assisted Al_2_O_3_ nanofluid flooding.

Applied EM Frequency (MHz)	Residual Retention Factor		Flooding Case
	NF	WF2	
**18.8**	1.090	0.944	α-Al_2_O_3_
	1.028	0.972	κ-Al_2_O_3_
	1.058	1.00	θ-Al_2_O_3_
	1.088	0.918	γ-Al_2_O_3_
167	1.212	0.945	α-Al_2_O_3_
	1.088	0.972	κ-Al_2_O_3_
	1.181	1.025	θ-Al_2_O_3_
	1.187	0.921	γ-Al_2_O_3_

## Appendix A

A typical Malaysian field, such as Tapis, has been chosen for a reference, with a well spacing of 1000–3000 ft. Based on the well spacing, the scale factor calculation has been performed to determine the optimum frequency to perform the EM-assisted nanoflooding experiments. Full-scale (*d_fs_*, ft) and the laboratory-scale (*d_lab_*, ft) dimension ratios are related by *n* scale factor as by,

(A1) n=dfsdlab

For the frequency (*f*, Hz) and wavelength (*λ*, m),

(A2) $$f_{lab}=\;n^{2}f_{fs}$$

(A3) $$\lambda =\;2\pi \sqrt{\frac{2}{\mu \sigma \omega }}$$

where *µ* = *µ*_0_ = 4*π* × 10^−7^ (V.s)/(A.m), *ω* = 2 *π* *f*, and *σ* = 12 S/m for sea water conductivity at 95 °C.
